# Immunology and psychosis: 22q11.2 syndrome as a model of study

**DOI:** 10.1192/j.eurpsy.2022.1803

**Published:** 2022-09-01

**Authors:** C. Garcia-Bernal, M.R. El-Idrissi Benhnia

**Affiliations:** 1 University Hospital Virgen del Rocio, Psychiatry, Seville, Spain; 2 Investigation Institute of Biomedicine of Seville, Biochemistry, Molecular Biology And Immunology, Seville, Spain

**Keywords:** Digeorge syndrome, psychosis, schizophrenia, inflammation

## Abstract

**Introduction:**

The 22q11.2 deletion syndrome (22q11DS) defines a set of developmental abnormalities due to a loss of genetic material. Phenotypic expression is highly varied. The immunological alterations that present as a severe combined immunodeficiency and the neurodevelopmental alterations stand out, especially the psychotic symptoms. It is the best described genetic alteration for the development of psychosis, presenting up to 30% of patients with compatible symptoms, with various hypotheses that justify it.

**Objectives:**

General objective:

1. Justify that SD2q11 can be used as a model for human research on the origin of psychosis.

**Specific objectives:**

1. Describe the pro-inflammatory state present in SD2q11.

2. Describe differences at the immunological level in SD2q11 among those patients with presence of psychotic symptoms of those who do not present them.

3. Describe possible biomarkers.

**Methods:**

A systematic review of the literature in the last 5 years using electronic resources (PubMed and WOS) until June 2021 following the PRISMA recommendations.

**Results:**

Three original articles were reviewed. There is a very marked pro-inflammatory state in 22q11DS patients with psychotic symptoms. They present an increase in subpopulations of CD4. The IL-17 is important in the formation in primitive stages of the hippocampus, its ease of breaking the blood-brain barrier (BBB). The neutrophil / lymphocyte ratio is presented as a possible biomarker to predict patients at high risk of developing psychosis.

**Conclusions:**

There is sufficient evidence that patients with 22q11DS can be used as a research model regarding possible hypotheses about the genesis of psychosis.

**Disclosure:**

No significant relationships.

